# Pneumothorax after Shoulder Arthroscopy: A Case Report

**DOI:** 10.5704/MOJ.2407.013

**Published:** 2024-07

**Authors:** FBN Tan, GWK Ho, EL Liow, LY Tan, SWL Ho

**Affiliations:** 1 Department of Orthopaedic Surgery, Tan Tock Seng Hospital, Singapore; 2 Department of Anaesthesiology, Tan Tock Seng Hospital, Singapore

**Keywords:** rotator cuff repair, decompression, pressure, complication

## Abstract

Shoulder arthroscopy is an increasingly common procedure. Pneumothorax post-shoulder arthroscopy is a rare complication. Our aim is to highlight a case report of pneumothorax post-shoulder arthroscopy and to conduct a literature review to evaluate the possible risk factors. We report the case of a 75-year-old male non-smoker, who underwent right shoulder arthroscopy without regional anaesthesia in the left lateral position and subsequently suffered a pneumothorax post-operatively. A PubMed Medline and Cochrane database search was carried out, and 32 articles were identified and thoroughly reviewed. Overall, among the articles that propose a mechanism, 75% (9/12) consider the pathogenesis to be multifactorial. The exact mechanism is currently unknown. Awareness of this complication and timely recognition are important to prevent life-threatening sequelae. Surgeons should maintain a low threshold for obtaining diagnostic plain radiographs in the event of clinical suspicion.

## Introduction

Shoulder arthroscopy is a common orthopaedic procedure. Pneumothorax, which is the accumulation of gas in the pleural space, is a rare complication of shoulder arthroscopy. There is limited existing literature regarding pneumothorax after arthroscopic shoulder procedures and is largely limited to case reports or small case series. There are many proposed mechanisms of pneumothorax post-arthroscopic surgery. However, it is difficult to determine the main cause of this complication. The purpose of this report and mini literature review is to highlight a case of pneumothorax after shoulder arthroscopy and to analyse the literature for possible risk factors.

**Fig. 1:**
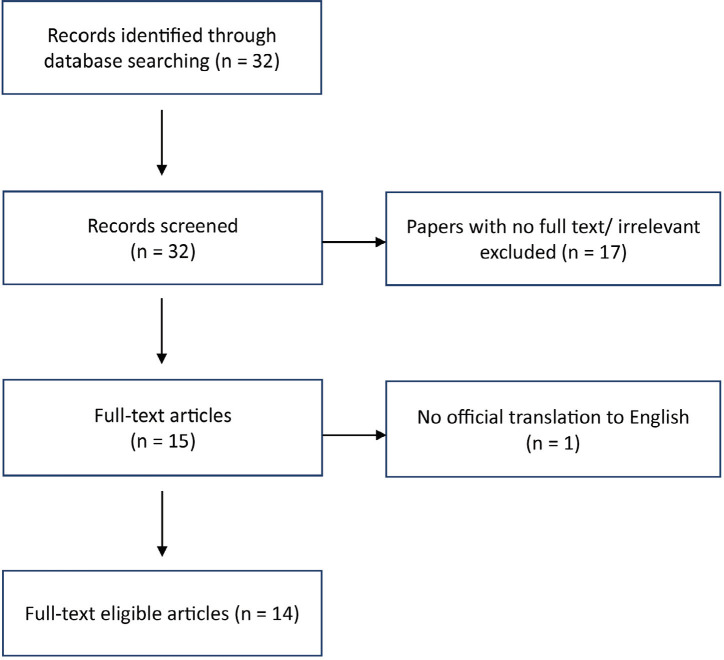
Flow diagram of article selection.

A systematic search was performed on PubMed Medline and Cochrane database from the date of database establishment till 01 June 2022. The search was carried out using the terms *shoulder arthroscopy, pneumothorax, pneumomediastinum, and subcutaneous emphysema.* The inclusion criteria were (1) full-text papers and (2) English language or English-translated articles. We obtained 32 papers during the initial search (Fig. 1). We excluded papers that were irrelevant (papers with no case study) and those lacking official translation into English. A total of 32 papers were thoroughly reviewed and 14 articles were found after inclusion and exclusion criteria were applied. We identified 20 cases of pneumothorax post-shoulder arthroscopy.

## Case Report

A 75-year-old man was reviewed in the orthopaedic surgery outpatient clinic with the primary complaint of atraumatic, persistent right shoulder pain for 2 years duration. The patient was a non-smoker and had a significant medical history of Parkinson’s disease, previous spontaneous intracranial haemorrhage, peptic ulcer disease, hypertension, and hyperlipidaemia. Physical examination of the right shoulder revealed a decreased range of motion of 110° of forward elevation and external rotation of 30°. There was a clinical weakness of the supraspinatus, infraspinatus, and subscapularis. Magnetic resonance imaging (MRI) of the right shoulder revealed a full-thickness complete tear of the supraspinatus tendon with retraction of the tendon fibres to the level of the glenohumeral joint. Fatty infiltration of the supraspinatus muscle (Goutallier grade III) was also noted. The infraspinatus and subscapularis tendons were also torn. In view of non-response to physiotherapy, the patient was offered surgical treatment. Arthroplasty was offered to the patient as a first-line surgical treatment, but the patient declined and opted for an arthroscopic rotator cuff repair. There were no outstanding medical issues noted at his preoperative anaesthesia review and the pre-operative chest radiograph showed mild bilateral lower zone atelectasis. There were no bullae observed.

The surgery was performed under intravenous induction of general anaesthesia with neuromuscular blockade. No upper extremity nerve block was performed. The patient was placed in left lateral decubitus position with the right upper limb abducted on continuous axial traction. Maintenance of anaesthesia was with Sevoflurane. The patient remained haemodynamically stable with oxygen saturation maintaining above 95% and peak inspiratory pressures between 14-17cm H2O throughout surgery.

The arthroscopic cuff repair was performed using five arthroscopic portals (posterior, anterior, lateral, posterolateral, and anterolateral). Repair of the subscapularis and a partial rotator cuff repair of the infraspinatus and supraspinatus was performed in combination with a biceps rerouting procedure. A subacromial decompression was also performed. The arthroscopic pressure was maintained at 40 mmHg. The surgical time was 3 hours 55 mins.

**Fig. 2:**
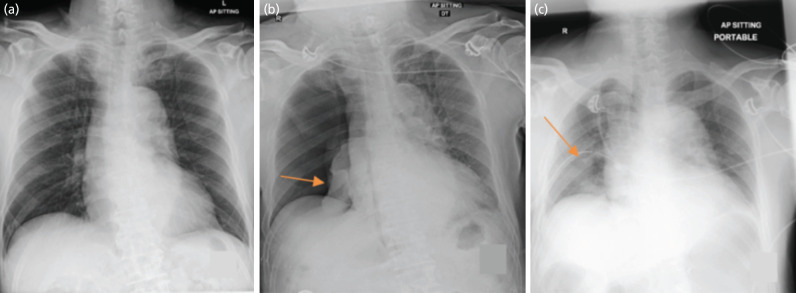
(a) Pre-operative chest radiograph – normal lung field expansion with no visible bullae noted. (b) Post-operative chest radiograph – right sided pneumothorax seen (indicated by arrow). (c) Chest radiograph post chest tube insertion (indicated by arrow) – with resolution of right sided pneumothorax seen.

The patient was extubated uneventfully and was noted to be saturating at 99% on room air. Upon arrival at the Post-Anaesthesia Care Unit (PACU), he desaturated to 92% on room air and oxygen supplementation was provided. Over the course of the next 20 minutes, the patient required increasing oxygen supplementation and was noted to have an oxygen saturation of 92% despite being on high flow nasal oxygen therapy of 60L/60%. The heart rate and blood pressure remained stable throughout the surgery. On examination, there was reduced air entry on the right chest. There was no tracheal deviation. Arterial blood gas showed type 1 respiratory failure (partial pressure of oxygen was 66mmHg and partial pressure of carbon dioxide was 38.5mmHg on 1.0 fraction of inspired oxygen). A chest radiograph was performed which revealed a large right-sided pneumothorax measuring 7cm at the hilum (Fig. 2). A 20 French chest drain was inserted into the right chest immediately. Subsequent surveillance radiographs showed complete resolution of the pneumothorax. The chest drain was removed five days later, and the patient made a full recovery.

## Discussion

Shoulder arthroscopy is an increasingly common surgical procedure for a variety of shoulder pathologies. Proposed advantages of arthroscopy over open surgery include smaller incisions, decreased bleeding, reduced post-operative pain, and decreased rates of post-operative stiffness1. Arthroscopic shoulder surgery can have potential complications. Complications post arthroscopic shoulder surgery include persistent pain, infection, and neurovascular injury2. Pneumothorax as a result of isolated interscalene brachial plexus blocks has been reported (Table I). However, there is also published literature on pneumothorax post-shoulder arthroscopy in the absence of regional anaesthesia (Table I). Additionally, although surgical factors such as patient positioning, arthroscopic techniques, equipment, and patient factors such as smoking and underlying respiratory conditions are considered to be significant, there is currently no consensus on any established risk factors for the development of pneumothorax post-shoulder arthroscopy. There are multiple cases which reported regional anaesthesia, particularly interscalene nerve blocks, to be associated with pneumothorax.

**Table I:** Summary of 14 case reports with 20 cases of pneumothorax post-shoulder arthroscopy.

Study	No. of cases	Type of anaesthesia	Symptom atic	Age	Smoker	Existing lung condition	Position	Ipsilateral to side of surgery	Diagnosed	Joint space entered	Proposed mechanism
Bowden BD, Williams WA, Stumpo LA, Stephens SP, DeCoons RM. Large asymptomatic pneumothorax following arthroscopic-assisted acromioclavicular joint reconstruction after ultrasound-guided interscalene block: a case report. JSES Int. 2020; 4(3): 551-4. doi: 10.1016/j.jseint.2020.02.013	1	GA, RA	No	30	No	No	Beach	Yes	Post-operatively	Glenohumeral Joint	Accidental pleural puncture during nerve block
Oldman M, Peng Pi P. Pneumothorax after shoulder arthroscopy: Don't blame it on regional anesthesia. Reg Anesth Pain Med. 2004; 29(4): 382-3. doi: 10.1016/j.rapm.2004.04.002	1	GA	Yes	41	No	No	-	-	Post-operatively	-	-
Bamps S, Renson D, Nijs S, Sermon. Pneumothorax after Shoulder Arthroscopy: A Rare but Life-threatening Complication. J Orthop Case Rep. 2016; 6(4): 3-5. doi: 10.13107/jocr.2250-0685.542	1	GA	Yes	42	No	No	Lateral decubitus	Yes	Post-operatively	Subacromial space	Rupture of parietal pleural
Dietzel DP, Ciullo JV. Spontaneous pneumothorax after shoulder arthroscopy: a report of four cases. Arthroscopy. 1996; 12(1): 99-102. doi: 10.1016/s0749-8063(96)90228-5	4	GA	-	22, 34, 37, 38	Yes	Yes	Lateral decubitus	-	Post-operatively	-	Rupture of blebs/bullae from lung disease
Lee HC, Dewan N, Crosby L. Subcutaneous emphysema, pneumomediastinum, and potentially life-threatening tension pneumothorax. Pulmonary complications from arthroscopic shoulder decompression. Chest. 1992; 101(5): 1265-7. doi: 10.1378/chest.101.5.1265	3	GA	Yes	-	No	No	Beach	-	Post-operatively	Subacromial space	Arthroscopic pump and shaver system causing pressure changes hence air entering surrounding soft tissues
Calvisi V, Lupparelli S, Rossetti S. Subcutaneous emphysema and pneumomediastinum following shoulder arthroscopy with brachial plexus block: a case report and review of the literature. Arch Orthop Trauma Surg. 2009; 129(3): 349-52. doi: 10.1007/s00402-008-0593-y	1	GA, RA	Yes	-	No	No	-	-	Intra-operatively	Subacromial space	Accidental pleural puncture nerve block, Bernoulli effect
Tang D, Liu Q, Chen C, Zhu W. Pneumothorax after shoulder arthroscopy: a case report and literature review. JSES Rev Rep Tech. 2021; 1(3): 194-7. doi: 10.1016/j.xrrt.2021.04.003	2	GA	Yes No	24, 60	Yes, No	No, No	Lateral decubitus	Yes	Post-operatively	-	Multifactorial (iatrogenic, traction, age, smoking, lung disease, trauma)
Kim JB, Choi MK, Jeon YK, Lee JM. Chest wall swelling and pneumothorax after shoulder arthroscopy: Were the 2 events totally independent? Medicine (Baltimore). 2017; 96(21): e7020. doi: 10.1097/MD.0000000000007020	1	GA	Yes	51	No	No	-	Yes	Post-operatively	-	High irrigating fluid pressure and volume may cause disruption of connective tissue
Leander-Olsson O, Borglund-Hemph A, Jakobsson JG. Pneumothorax following shoulder arthroscopy under combined regional and general anaesthesia-A case report. Int J Surg Case Rep. 2016; 24: 73-6. doi: 10.1016/j.ijscr.2016.05.012	1	GA, RA	Yes	72	Yes (ex-smoker)	Yes	Beach	No	Post-operatively	-	Accidental pleural puncture, barotrauma
Lin YJ, Chen GX, Zhang Y. Postoperative management of spontaneous pneumothorax in arthroscopic shoulder superior capsular reconstruction: A case report and review of literature. Chin J Traumatol. 2022; 25(3): 181-3. doi: 10.1016/j.cjtee. 2022.03.005	1	GA	Yes	66	No	No	-	Yes	Intra-operatively	Subacromial space	No proposed mechanism
Li R, Lall A, Lai E, Gruson Kl. Tension Pneumothorax After Ultrasound-Guided Interscalene Block and Shoulder Arthroscopy. Am J Orthop (Belle Mead NJ). 2015; 44(10): E407-10.	1	GA, RA	Yes	56	No	No	Beach	Yes	Intra-operatively	Subacromial space	Multifactorial (anaesthesia, surgical, patient)
Cassone MA, Kish KL, Nester JR, Hoffman LM. Case Report and Literature Review: Post-Arthroscopy Pneumothorax with Anterior Decompression. Clin Pract Cases Emerg Med. 2020; 4(4): 580-3. doi: 10.5811/cpcem.2020.8.48618	1	GA	Yes	60	No	No	Lateral decubitus	Yes	Post-operatively	Subacromial space	Multifactorial (anaesthesia, surgical, patient)
Shariati MJ, Kachooei AR, Ebrahimzadeh MH. Massive Emphysema and Pneumothorax Following Shoulder Arthroscopy under General Anaesthesia: A Case Report. Arch Bone Jt Surg. 2017; 5(6): 459-63. doi: 10.22038/abjs.2017.23435.1620	1	GA	Yes	61	No	No	Beach	Yes	Post-operatively	-	Barotrauma, trauma by arthroscopy equipment
Niu WY, Liao YA, Liu SC, Liu YC, Lin FS. Air- Driven Drill Induced Diffuse Subcutaneous Emphysema and Pneumothorax During Shoulder Arthroscopic Surgery. Asian J Anesthesiol. 2020; 58(1): 50-3. doi: 10.6859/aja.202003-58(1).0007	1	GA, RA	Yes	53	No	No	Lateral decubitus	Yes	Intra-operatively	-	Arthroscopic pump and shaver system causing pressure changes hence air entering surrounding soft tissues

The incidence of pneumothorax after interscalene nerve block was found to be 0.2%-3%3. Bowden *et al* (Table I) described a 30-year-old female who underwent arthroscopic assisted acromioclavicular joint reconstruction with an interscalene block administered. Being a non-smoker with no previous lung condition, the authors postulated that there was an accidental pleural puncture during nerve block. Leander-Olsson *et al* (Table I) reported a case of a 72-year-old male who was found to have a large pneumothorax while undergoing left shoulder arthroscopy. Leander-Olsson *et al* (Table I) also attributed the pneumothorax to needle injuries during upper extremity blocks, but also cited external pressure on the upper pleura dome, thermal forces induced by diathermia, and barotrauma during intubation to be other contributing factors. Even under ultrasound guidance, certain factors such as loss of visualisation of the needle tip, probe, and plane and hydro-visualisation as well as patient factors such as lung disease, smoking, emphysema, and obesity can increase the risk associated with pneumothorax associated with ultrasound-guided upper extremity blocks. A recurring argument in these cases is that of pleural puncture. Although the patients in these case reports had both GA and RA, the authors mostly highlight regional anaesthesia as the main cause of pneumothorax. However, it is important to note that our patient, as well as 13 out of 20 (65.0%) of the cases of post-operative pneumothorax only underwent general anaesthesia. As such, it is difficult to solely attribute post-operative pneumothorax to regional anaesthesia alone. Under general anaesthesia, airway forces, bronchus intubation and patient risk factors such as smoking, and lung disease could contribute to post-operative pneumothorax. Additionally, high trans-pulmonary pressures during positive pressure ventilation, difficult intubation or use of airway catheters can also lead to barotrauma causing iatrogenic pneumothorax. Barotrauma associated with GA is rare, with the incidence quoted to be as low at 0.5%4. Although regional anaesthesia might result in direct pleural injury, patients undergoing shoulder arthroscopy under general anaesthesia alone may also develop post-operative pneumothorax.

Shoulder arthroscopy can be performed in either the beach chair or lateral decubitus position. In the case reports that did state patient positioning, 9 out of 16 (56.3%) of cases were in the lateral decubitus position. Although the lateral decubitus position allows for better visualisation and instrument access, complications associated with the lateral decubitus position include traction injury and difficult airway management. Tang *et al* (Table I) also proposed that the continuous axial traction in lateral decubitus position could potentially increase laxity of the junction around shoulder and chest wall and result in a pneumothorax. Niu *et al* (Table I) mentioned that decubitus position may cause more subcutaneous emphysema due to effects of gravity and, hence causing pneumothorax. Dietzel *et al* (Table I) described four cases of pneumothorax post arthroscopy of which all four patients were in lateral decubitus position. They posited that the pressure gradient from positive pressure of anaesthesia with the ipsilateral lung being the highest in lateral decubitus increases the chance of bleb/pleura rupture. However, these four patients were smokers which could possibly mean the pleural dome is higher than non-smokers due to hyperinflated lungs caused by smoking.

Ten out of 11 of our case reports were diagnosed with pneumothorax on the ipsilateral side of the shoulder arthroscopy (Table I). This may suggest that a combination of factors such as smoking, arthroscopic techniques, and positioning may be more significant in terms of risk factors compared to general anaesthesia alone.

In the absence of such factors, arthroscopic equipment has also been found to cause post-operative pneumothorax. Lee *et al* (Table I) proposed that surgical-related external forces from arthroscopic shavers, irrigation fluid, and infusion pumps were responsible for causing the pneumothoraxes in the three patients he reported. As for the mechanism of injury, the authors suggested that the change of pressure in subacromial space may force air into surrounding tissues and result in a pneumothorax. This occurs when air is drawn through the anterior arthroscopic portal when the suction is turned off as well as the positive pressure from the pump. Notably, only one out of all the papers in the literature review stated the arthroscopic pressures used. Calvisi *et al* (Table I) also proposed a similar mechanism. The authors mentioned that the arthroscopic pump, high-section shaver, and outflow cannula caused the Bernoulli effect. This is especially so if the subacromial space, which does not have a capsule compared to the glenohumeral joint, has been entered which allows for air or fluid extravasation. Other factors which have been described in fluid extravasation during shoulder arthroscopy include resection of the glenohumeral joint capsule, length of surgery, the volume of irrigation fluid, and iatrogenic tears or lesions of the rotator cuff and deltoid muscles. These factors may also be significant but were not mentioned in the case reports. It would be worthwhile to look into all these factors since arthroscopic pressures and techniques might be an important risk factor for developing post-operative pneumothorax.

Smoking and pre-existing respiratory comorbidities have been identified to be significant risk factors. In the four cases of spontaneous pneumothorax after shoulder arthroscopy that Dietzel *et al* (Table I) shared, all patients had a significant history of either smoking or asthma. The authors attributed the underlying cause to be due to the spontaneous rupture of underlying bullae. Even though they drew such a conclusion, it is important to note that 14 out of 20 (70.0%) of cases in our literature review had no history of smoking or respiratory comorbidity. As such, the presence of pre-existing respiratory disease may be unlikely to be a common cause of pneumothorax post arthroscopy.

Other patient factors such as obesity, older age, looser subcutaneous soft tissue, and anatomical variants which may lead to aberrant connections between the glenohumeral joint and surrounding soft tissues have also been described to increase the risk of fluid extravasation during shoulder arthroscopy. These factors may also be relevant in air extravasation which may lead to pneumothorax.

Our patient had a significant past medical history of Parkinson's disease (PD) with suspected primary progressive gait. Patients with PD may suffer from recurrent falls, complications of immobility, depression, dementia, and sleep disturbances. The prevalence and incidence of respiratory dysfunction are not well defined, but it has been reported that PD has been linked to obstructive and restrictive pulmonary disease.

Our patient did not have pre-existing lung conditions and was asymptomatic to the day of the operation. Hovestadt *et al* concluded that lung function tests conducted on PD patients without any clinical signs or symptoms of pulmonary disease suggest subclinical upper airway obstruction5. Although other operative factors seem to have stronger evidence, it is possible that PD was a risk factor for pneumothorax considering chronic obstructive lung disease is a known risk factor for spontaneous pneumothorax.

Sixteen out of 20 (80.0%) of the cases in the literature review were diagnosed post-operatively and 4 out of 20 cases were diagnosed intra-operatively. Most of the pneumothoraxes were diagnosed shortly after the patient had symptoms or oxygen desaturation. Time to diagnosis tends to be early post-operatively rather than a delayed diagnosis of more than 24 hours. Eighty-eight percent of the patients in this review were symptomatic from pneumothorax. However, it is important to note that there were some cases of asymptomatic pneumothorax. Surgeons should have a low threshold to attain a chest radiograph in high-risk patients with respiratory distress symptoms. Timely recognition allows for rapid diagnosis and intervention. All patients in this series had clinical resolution of the pneumothorax.

Pneumothorax post-shoulder arthroscopy is a rare complication. The exact mechanism is currently unknown. Awareness of this complication and timely recognition are important to prevent life-threatening sequelae. Surgeons should have a low threshold to obtain diagnostic plain radiographs in the event of clinical suspicion.
